# Association of the Composite Dietary Antioxidant Index with All‐Cause, Cancer, and Cardiovascular Mortality: A Prospective Cohort Study

**DOI:** 10.1002/fsn3.71351

**Published:** 2026-01-14

**Authors:** Niloufar Abdollahpour, Nadia Homayounfar, Farima Farsi, Sara Saffar Soflaei, Mohammad Kalate Rahmani, Mina Mousavi, Mohsen Mouhebati, Habibollah Esmaily, Najmeh Seifi, Majid Ghayour‐Mobarhan

**Affiliations:** ^1^ Department of Nutrition, Faculty of Medicine Mashhad University of Medical Sciences Mashhad Iran; ^2^ Student Research Committee Mashhad University of Medical Sciences Mashhad Iran; ^3^ Obesity and Eating Habits Research Center Tehran University of Medical Sciences Tehran Iran; ^4^ Metabolic Syndrome Research Center Mashhad University of Medical Sciences Mashhad Iran; ^5^ Department of Biostatistics, School of Health Mashhad University of Medical Sciences Mashhad Iran; ^6^ Social Determinants of Health Research Center Mashhad University of Medical Sciences Mashhad Iran

**Keywords:** all‐cause mortality, cancer mortality, cardiovascular disease, cardiovascular mortality, composite dietary antioxidant index, dietary antioxidant

## Abstract

Oxidative stress is a major contributor to chronic diseases such as cardiovascular disease (CVD) and cancer, and antioxidants may play a protective role. However, the link between overall dietary antioxidant intake, measured using the Composite Dietary Antioxidant Index (CDAI), and long‐term health outcomes remains unclear. This prospective cohort study investigated the association between CDAI and cause‐specific mortality, including CVD and cancer, as well as all‐cause mortality and CVD incidence, using data from 6484 adults (mean age: 48.38 years, 39.98% male) in the Mashhad Stroke and Heart Atherosclerosis Disorder (MASHAD) cohort. Dietary intake was evaluated using a validated food frequency questionnaire, and CDAI was calculated from six dietary antioxidants. Participants were followed for 10 years. Kaplan–Meier survival analysis showed that individuals in the third and fourth quartiles of CDAI had significantly lower cancer mortality (*p* = 0.011) and cardiovascular mortality (*p* = 0.021). Multivariable Cox regression revealed a significant inverse association between CDAI and cardiovascular mortality in the second (OR = 0.531; 95% CI: 0.391–0.884) and third (OR = 0.535; 95% CI: 0.321–0.892) quartiles, but not in the fourth (OR = 0.847; 95% CI: 0.537–1.337). Higher CDAI was also linked to lower cancer mortality in crude (OR = 0.424; 95% CI: 0.224–0.802), first (OR = 0.384; 95% CI: 0.198–0.747), and second (OR = 0.398; 95% CI: 0.202–0.785) models, though this association weakened after full adjustment (OR = 0.469; 95% CI: 0.165–1.331). No significant link was found between the highest CDAI quartile and all‐cause mortality (*p* > 0.05). Overall, these findings suggest that moderate antioxidant intake may offer protective effects, particularly for cardiovascular outcomes.

## Graphical abstract

This prospective cohort study investigates the relationship between the Composite Dietary Antioxidant Index (CDAI) and cause‐specific as well as all‐cause mortality. Higher CDAI levels were significantly associated with reduced cardiovascular and cancer mortality, though associations weakened after full adjustment. The findings support the potential role of antioxidant‐rich diets in lowering long‐term health risks, particularly cardiovascular mortality.

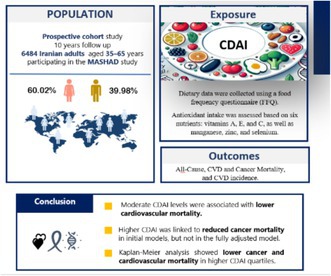

## 1 Introduction

Noncommunicable diseases (NCDs) are the leading cause of disability and death globally, with cardiovascular disease (CVD) and cancers accounting for the highest mortality rates (Peng et al. 2024; Taheri Soodejani 2024). CVDs contribute approximately one‐third of all deaths, while cancer accounts for roughly one in six deaths (Wang and Yi 2022; Consortium GCR 2023; Bray et al. 2024). In Iran, the situation is similar, with CVDs causing 46% of all deaths (Saki et al. 2022). Following CVDs and accidents, cancer ranks third in the cause of death in Iran (Izadi et al. 2022).

Studies have demonstrated that chronic diseases such as CVDs and cancers are closely linked to inflammatory status and oxidative stress, which arise from an imbalance between antioxidants and reactive oxygen species (Wang and Yi 2022; Townsend et al. 2022). Inflammation can exacerbate oxidative stress, creating a vicious cycle that triggers more inflammation. This interplay accelerates processes like atherosclerosis, contributing to CVD, and promotes conditions that support cancer cell survival (Ma, Zhou, et al. 2023; Dharshini et al. 2023).

Among effective strategies to mitigate these harmful effects, a diet that is rich in antioxidants—capable of neutralizing free radicals—stands out as a promising intervention to reduce both oxidative stress and inflammation. To assess and reflect the individual's dietary antioxidant profile, the Composite Dietary Antioxidant Index (CDAI) can be used (Ma, Zhou, et al. 2023; Teng et al. 2024). CDAI is a summary and composite score based on the six selected dietary antioxidant vitamins and minerals, including selenium, manganese, zinc, and vitamins A, C, and E (Ma, Zhou, et al. 2023; Keshani et al. 2023; Liu et al. 2023). CDAI was designed and developed because of its association with inflammatory markers, such as IL‐1 and tumor necrosis factor‐α (TNF‐α), and its anti‐inflammatory effects on them (Wang and Yi 2022; Liu et al. 2023).

Previous researches have demonstrated a negative relationship between CDAI and outcomes such as CVD incidence, CVD deaths, and mortality from all causes (Wang and Yi 2022; Luo et al. 2024; Qin et al. 2024; Jiang and Shen 2025). In Iran, while several studies have investigated the relationship between dietary antioxidant intakes and either CVDs or mortality (Keshani et al. 2023; Mirmiran et al. 2022; Vahid et al. 2022), to our knowledge, none have specifically focused on the association between CDAI and all‐cause or specific‐cause mortality. Given the high prevalence of NCDs and their significant mortality rates in Iran, a large population‐based study that considers regional dietary habits and cultural factors is essential. This study aims to provide valuable insights into the associations between CDAI and key health outcomes. Specifically, it seeks to investigate the relationship between CDAI and the incidence of cardiovascular disease, as well as all‐cause, CVD‐related, and cancer mortality within a substantial population in Iran. Such a comprehensive analysis can lead to evidence‐based, tailored preventative medical approaches to effectively reduce the burden of NCDs in Iran.

## 2 Methods

### 2.1 Study Design and Population

The population sample of this prospective cohort study was obtained from the Mashhad Stroke and Heart Atherosclerotic Disorder (MASHAD), which began in 2010 to study the association between CVDs and their risk factors. Details relevant to the design of the study have been reported thoroughly in a previously published article (Ghayour‐Mobarhan et al. 2015). Briefly, a total number of 9704 adults aged 35–65 years from the general urban population of Mashhad, Iran, were enrolled in the MASHAD study, and baseline data on demographic characteristics, dietary intakes, anthropometric and laboratory measurements, as well as blood pressure, were collected. For the present study, participants who reported energy intakes exceeding 4200 kcal and below 800 kcal, those with incomplete dietary intakes data, and pregnant or breastfeeding women were excluded. As a result, 6484 subjects remained in the study for final statistical analyses. The Human Research Ethics Committee of the Faculty of Medicine, Mashhad University of Medical Sciences (MUMS) approved the study protocol, and all individuals provided written informed consent (IR.MUMS.MEDICAL.REC.1395.458).

### 2.2 Anthropometric Measurements and Physical Activity Level

Standard protocols were implemented for anthropometric evaluations including weight and height. Body weight and height were measured for all participants of the first phase of the MASHAD study. Weight was assessed to the nearest 0.1 kg using a calibrated digital scale (Seca, Japan), while participants were wearing light clothing and no shoes. Height was determined to the nearest 0.1 cm using a stadiometer, with the participant's head positioned in the Frankfort horizontal plane (Ghayour‐Mobarhan et al. 2015). To compute BMI, we divided weight (kg) by height squared (m). A physical activity questionnaire, adapted from the Scottish Heart Health Study (SHHS)/MONICA questionnaire, focused on time spent on activities during work, nonwork time, and in bed (Bolton‐Smith et al. 1992). Total energy expenditure (TEE) was calculated by incorporating the gender‐specific integrated energy index (IEI) values—1.51, 2.49, and 4.34 for men and 1.61, 2.52, and 4.39 for women, corresponding to inactive, moderate, and active levels, respectively—along with the energy cost of each activity. Basal metabolic rate (BMR) was estimated using the FAO/WHO/UNU equation. Finally, PAL was computed as the ratio of TEE to BMR (Bahari et al. 2023).

### 2.3 Biochemical Measurements

After a 14‐h overnight fast, 10 mL of venous blood was taken from each participant and was subsequently centrifuged for 30 min at room temperature in order to isolate the serum or plasma. Serum concentrations of total low‐density lipoprotein‐cholesterol (LDL‐C), cholesterol, triglyceride (TG), high‐density lipoprotein‐cholesterol (HDL‐C), and fasting plasma glucose (FPG) were assessed utilizing a BT‐3000 autoanalyzer (Biothecnica, Italy) with commercial kits from Pars Azmoon (Tehran, Iran). More details have been described previously (Ghayour‐Mobarhan et al. 2015). To define chronic disease, clinical and biochemical data were used as follows: hypertension was determined by systolic blood pressure (SBP) ≥ 140 mmHg or a diastolic blood pressure (DBP) ≥ 90 or treatment of previously diagnosed hypertension (HTN), and dyslipidemia (LDL‐C ≥ 130, or TGs ≥ 150, or a HDL‐C < 40 mg/dL in men and < 50 mg/dL in women) or previously treated diagnosed dyslipidemia (Ghayour‐Mobarhan et al. 2015).

### 2.4 Food‐Frequency Questionnaire

For the assessment of dietary intake of participants, a validated semi‐quantitative food frequency questionnaire (FFQ) of 65 items was used in the first phase of the MASHAD study (Ahmadnezhad et al. 2017). This questionnaire includes five frequencies of use (per day, week, month, rarely, and never) and household or standard scales for portion size of all food items (Ghayour‐Mobarhan et al. 2015). To analyze food compositions, the Dietplan 6 software (Forestfield Software Ltd., Horsham, West Sussex, UK) was used. The Diet Quality Index‐International (DQI‐I) was used to assess overall diet quality, based on four components: variety, adequacy, moderation, and overall dietary balance. Variety was evaluated based on the diversity of food groups and protein sources, with a score ranging from 0 to 20. Adequacy reflected sufficient intake of grains, fruits, and vegetables, fiber, protein, iron, calcium, and vitamin C, which was scored from 0 to 40. Moderation assessed limitations in cholesterol, saturated and total fat, sodium, and empty‐calorie foods, with scores from 0 to 30. Overall balance captured the macronutrient and fatty acid ratio, contributing up to 10 points. The total DQI‐I score ranged from 0 to 100, with higher scores representing better diet quality (Kim et al. 2003).

### 2.5 Composition Dietary Antioxidant Index Calculation

To calculate the Composite Dietary Antioxidant Index (CDAI) in our dataset, the intakes of six dietary vitamins and minerals, including vitamins A, C, and E, selenium, manganese, and zinc, were standardized by subtracting the mean and dividing it by the standard deviation specific to the population. The CDAI was then computed by summing the standardized nutrient intakes, assigning equal weight to each nutrient (Wright et al. 2004).

### 2.6 Follow Up and Outcomes

The second phase of the MASHAD study commenced in 2018 with follow‐up of participants from the first phase for almost a decade. Some of the initial participants did not enter the second phase of the study due to reasons such as migration, unwillingness, unavailability, and death. Ultimately, 9404 individuals participated in the second phase. The probable incidence of CVD events was assessed in all subjects in the second phase of the study with a view to previous medical examinations and clinical tests as well as the judgments of three cardiologists and one neurologist. If necessary, a panel of cardiologists decided on the participants' final diagnoses. The individuals in our study were followed up for all‐cause mortality, cancer, and CVD mortality, and CVD incidence. The CVD mortality includes fatalities from myocardial infarction (MI), cerebrovascular accident, chronic ischemic heart disease, arrhythmia, peripheral vascular disease (PVD), pulmonary thromboembolism, and cardiac arrest. Cancer mortality was defined as any death resulting from different types of cancers, verified by death certificates or family‐reported cause‐of‐death surveys completed by immediate family members.

### 2.7 Statistical Analysis

In the present study, statistical analysis was performed employing the Statistical Package for the Social Sciences (SPSS, Chicago, USA) version 25.0 and RStudio (RStudio, PBC, Boston, MA, USA) version 4.1.2. Continuous variables were reported in mean ± SD format and categorical ones were reported in frequency (%). One‐way ANOVA and *χ* ^2^ test were utilized to analyze the continuous variables and categorical variables respectively. Also, in case of abnormal or skewed distribution, the Kruskal–Wallis *H* test was employed to test the variations across the different quartiles of CDAI. The calculated CDAI values were then categorized into quartiles (Q1–Q4). The cutoff points defining these quartiles were as follows: Q1 (< −2.56), Q2 (−2.56 to −0.42), Q3 (−0.42 to 1.98), and Q4 (> 1.98). The Kolmogorov–Smirnov test was utilized to check variable‐distribution normality. Cox Regression Analysis was applied to assess the connection between CDAI quartiles and the risk of mortality including CVD mortality, cancer mortality, and mortality resulting from all other causes, while the effect of age, sex, BMI, marriage status, smoking status, PAL, educational level, energy intake, DQI‐I, hypertension, dyslipidemia, and diabetes were controlled. Furthermore, Cox regression models were used to assess the associations between quartiles of the CDAI and the incidence of CVDs. Analyses were performed using three models: a crude model; Model 1, adjusted for age and sex; Model 2, further adjusted for body mass index (BMI), marital status, smoking status, physical activity level, and educational level; and Model 3, additionally adjusted for energy intake, the DQI‐I score, hypertension, dyslipidemia, and diabetes. The associations between the CDAI quartiles and mortality or CVD outcomes were evaluated using stratified Cox proportional hazards models, with the first quartile (Q1) serving as the reference category. Additionally, Spearman's rank correlation coefficients were used to assess the relationships between the CDAI and key variables (including energy intake, DQI‐I score, age, and physical activity level) and visualized using scatter plots. Restricted Cubic Spline (RCS) was used to explore the nonlinear relationship between CDAI and all‐cause mortality, CVD, and cancer mortality, as well as CVD incidence. Kaplan–Meier survival curves were used to show the outcomes according to CDAI quartiles. *p*‐values below 0.05 were taken as statistically significant.

## 3 Results

**FIGURE 1:**
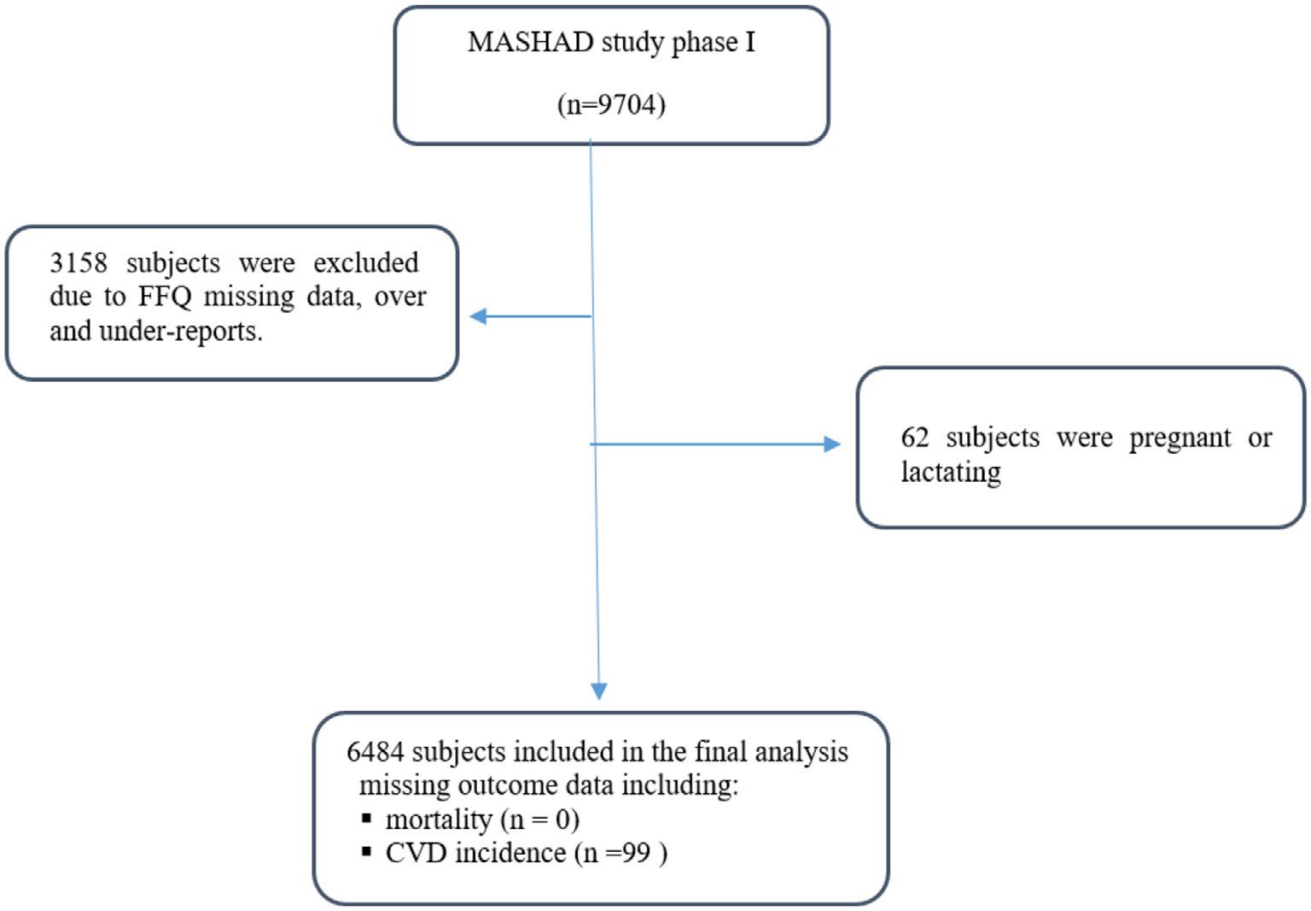
Flow diagram of study subject subgroups analyses. CVD, cardiovascular disease; FFQ, food frequency questionnaire.

This study incorporated 6484 participants (39.98% male) with an average age of 48.38 years. The flow diagram detailing the selection of participants is presented in Figure 1. Demographic characteristics according to CDAI quartiles are presented in Table 1. Over the follow‐up period, 311 deaths were reported, including 138 incidences from CVD and 87 deaths from cancer. Additionally, 751 new cases of cardiovascular disease were reported. Significant differences were observed in age (*p* < 0.001), physical activity levels (*p* < 0.001), marital status (*p* < 0.001), employment status (*p* < 0.001), education level (*p* < 0.001), smoking status (*p* = 0.023), and energy intake (*p* < 0.001) among CDAI quartiles. Participants in higher quartiles were generally younger, more likely to have a university degree, be married, employed, and current smokers, and have higher energy intake but tended to have lower physical activity levels. Considering other health‐related factors, hypertension prevalence (*p* = 0.013) and CVD‐related mortality (*p* = 0.016) varied significantly across quartiles. However, diabetes (*p* = 0.195), dyslipidemia (*p* = 0.915), CVD incidence (*p* = 0.218), and all‐cause mortality (*p* = 0.671) showed no significant differences among quartiles.

**TABLE 1:** Demographic characteristics according to CDAI quartiles.

Variables	CDAI				
	Q1 (*N* = 1620)	Q2 (*N* = 1614)	Q3 (*N* = 1624)	Q4 (*N* = 1626)	*p*
	< −2.56	−2.56 to 0.42	−0.42 to 1.98	1.98 <	
Age (year)^a^	49.15 ± 8.42	48.28 ± 8.15	48.44 ± 8.16	47.67 ± 8.02	< 0.001
Male, *N* (%)	448 (27.65)	591 (36.61)	717 (44.15)	848 (52.15)	< 0.001
Physical activity^a^	1.66 ± 0.29	1.61 ± 0.28	1.59 ± 0.29	1.57 ± 0.30	< 0.001
Marriage status, *N* (%)					
Single	16 (0.98)	5 (0.31)	9 (0.55)	12 (0.74)	< 0.001
Married	1445 (89.20)	1506 (93.31)	1537 (94.64)	1544 (94.96)	
Divorced	32 (1.98)	23 (1.43)	18 (1.11)	18 (1.11)	
Widow	127 (7.84)	80 (4.96)	60 (3.69)	52 (3.20)	
Employment status, *N* (%)					
Student	3 (0.19)	5 (0.31)	3 (0.18)	8 (0.49)	< 0.001
Employee	481 (29.69)	556 (34.45)	624 (38.42)	764 (46.99)	
Unemployed	1016 (62.72)	894 (55.39)	783 (48.21)	658 (40.47)	
Retried	118 (7.28)	159 (9.85)	214 (13.18)	195 (11.99)	
Educational level, *N* (%)					
Up to Diploma	1529 (94.38)	1459 (90.40)	1416 (87.19)	1388 (85.47)	< 0.001
Some college or university educated	91 (5.62)	155 (9.60)	208 (12.81)	238 (14.66)	
Smoking status, *N* (%)					
Nonsmoker	1148 (70.86)	1133 (70.20)	1133 (69.77)	1065 (65.50)	0.023
Former‐smoker	157 (9.69)	145 (8.98)	161 (9.91)	179 (11.01)	
Current‐smoker	315 (19.44)	336 (20.82)	330 (20.32)	382 (23.49)	
BMI (kg/m^2^)^a^	28.05 ± 4.84	28.01 ± 4.71	27.93 ± 4.60	27.91 ± 4.74	0.826
Energy intake (kcal/day)^a^	1435.92 ± 293.36	1817.87 ± 308.01	2130.46 ± 339.05	2667.98 ± 511.11	< 0.001
HTN, *N* (%)	183 (11.30)	159 (9.85)	165 (10.16)	137	0.013
Diabetes, *N* (%)	254 (15.68)	249 (15.43)	226 (13.92)	217	0.195
Dyslipidemia, *N* (%)	185 (11.42)	188 (11.65)	204 (12.56)	196	0.915
Incidence of CVD, *N* (%)	182 (12.83)	176 (12.41)	181 (12.76)	212 (15.17)	0.218
CVD mortality, *N* (%)	41 (2.53)	26 (1.61)	27 (1.66)	44 (2.70)	0.016
All‐cause mortality, *N* (%)	83 (5.12)	80 (4.96)	69 (4.25)	79 (4.86)	0.671
Cancer mortality, *N* (%)	28 (38.35)	21 (33.87)	23 (25)	15 (17.86)	0.022
DQI‐I^a^	55.14 ± 7.20	61.65 ± 6.21	64.70 ± 6.49	65.42 ± 6.43	< 0.001
CDAI^a^	−4.21 ± 1.23	−1.48 ± 0.61	0.71 ± 0.70	4.98 ± 3.44	< 0.001

*Note:* Percentages are calculated in each CDAI quartile. A *p*‐value of less than 0.05 was considered statistically significant. ANOVA was applied to assess continuous variables, while categorical variables were evaluated using the chi‐square test.

Abbreviations: BMI, Body Mass Index; CDAI, Composite Dietary Antioxidant Index; CVD, Cardiovascular Disease; DQI‐I, Dietary Quality Index‐International; HTN, hypertension.

^a^ Values are stated as mean ± SD.

Table 2 shows the results of the cox regression analysis examining the relationship between CDAI and the risk of mortality and CVD incidence. In the crude model, no significant association was discovered between CDAI quartiles and all‐cause mortality (OR (95% CI): 0.915 (0.672–1.245)). Following adjustment for confounding factors in the subsequent models, the association remained nonsignificant (OR (95% CI): 1.003 (0.671–1.500) in the final model). The crude model revealed no significant association between CDAI quartiles and CVD mortality (OR (95% CI): 0.996 (0.647–1.533)). After adjusting for confounding variables in model 1 (OR (95% CI): 0.926 (0.597–1.435)) and model 2 (OR (95% CI): 0.920 (0.591–1.433)), the association remained nonsignificant. In the fully adjusted model, the association between cardiovascular mortality and CDAI was significant in the second (OR (95% CI): 0.531 (0.391–0.884)) and third (OR (95% CI): 0.535 (0.321–0.892)) quartiles, but this association was no longer significant in the fourth quartile (OR (95% CI): 0.847 (0.5371.337)).

**TABLE 2:** Cox regression analysis of the association between the composite dietary antioxidant index, mortality risk, and cardiovascular disease incidence.

Variables	CDAI				
	Q1 (*N* = 1620)	Q2 (*N* = 1614)	Q3 (*N* = 1624)	Q4 (*N* = 1626)	*p*
**All‐cause mortality**					
Crude Model	1	0.942 (0.693–1.281)	0.796 (0.578–1.096)	0.915 (0.672–1.245)	0.560
Model 1	1	0.967 (0.710–1.317)	0.736 (0.532–1.018)	0.920 (0.671–1.262)	0.257
Model 2	1	0.952 (0.699–1.298)	0.755 (0.544–1.046)	0.924 (0.673–1.270)	0.358
Model 3	1	1.045 (0.751–1.455)	0.818 (0.567–1.182)	1.003 (0.671–1.500)	0.488
**Cardiovascular disease mortality**					
Crude Model	1	0.542 (0.331–0.888)	0.640 (0.393–1.041)	0.996 (0.647–1.533)	0.025
Model 1	1	0.532 (0.325–0.871)	0.560 (0.339–0.924)	0.926 (0.597–1.435)	0.015
Model 2	1	0.528 (0.322–0.866)	0.551 (0.333–0.911)	0.920 (0.591–1.433)	0.015
Model 3	1	0.531 (0.319–0.884)	0.535 (0.321–0.892)	0.847 (0.537–1.337)	0.024
**Cancer mortality**					
Crude Model	1	0.990 (0.552–1.775)	0.611 (0.347–1.076)	0.424 (0.224–0.802)	0.022
Model 1	1	0.910 (0.499–1.660)	0.589 (0.327–1.062)	0.384 (0.198–0.747)	0.018
Model 2	1	0.935 (0.508–1.719)	0.568 (0.312–1.036)	0.398 (0.202–0.785)	0.023
Model 3	1	1.022 (0.501–2.083)	0.622 (0.274–1.414)	0.469 (0.165–1.331)	0.296
**Cardiovascular disease incidence**					
Crude Model	1	1.025 (0.833–1.262)	1.007 (0.820–1.238)	1.154 (0.946–1.407)	0.437
Model 1	1	1.037 (0.842–1.277)	0.944 (0.766–1.164)	1.164 (0.950–1.425)	0.206
Model 2	1	1.018 (0.826–1.254)	0.941 (0.763–1.161)	1.133 (0.924–1.390)	0.321
Model 3	1	1.036 (0.832–1.290)	0.896 (0.708–1.135)	1.053 (0.813–1.363)	0.433

*Note:* Model 1: adjusted by age, sex. Model 2: adjusted by model 1 + BMI, marriage status, smoking status, PAL, educational level. Model 3: adjusted by model 1 and 2 + energy intake, DQI‐I, hypertension, Dyslipidemia, Diabetes.

Regarding the risk of cancer death, in the crude model, higher CDAI quartiles were significantly associated with a reduced risk of cancer mortality (OR (95% CI): 0.424 (0.224–0.802)). After adjustments were made for confounding variables in the first (OR (95% CI): 0.384 (0.198–0.747)) and second (OR (95% CI): 0.398 (0.202–0.785)) models, the association was still significant. However, in the fully adjusted model, the association was no longer significant (OR (95% CI): 0.469 (0.165–1.331)). As for CVD occurrence, none of the crude or adjusted models demonstrated a significant association between CDAI and the incidence of CVD (OR (95% CI): 1.053 (0.813–1.363) in the final model).

**FIGURE 2:**
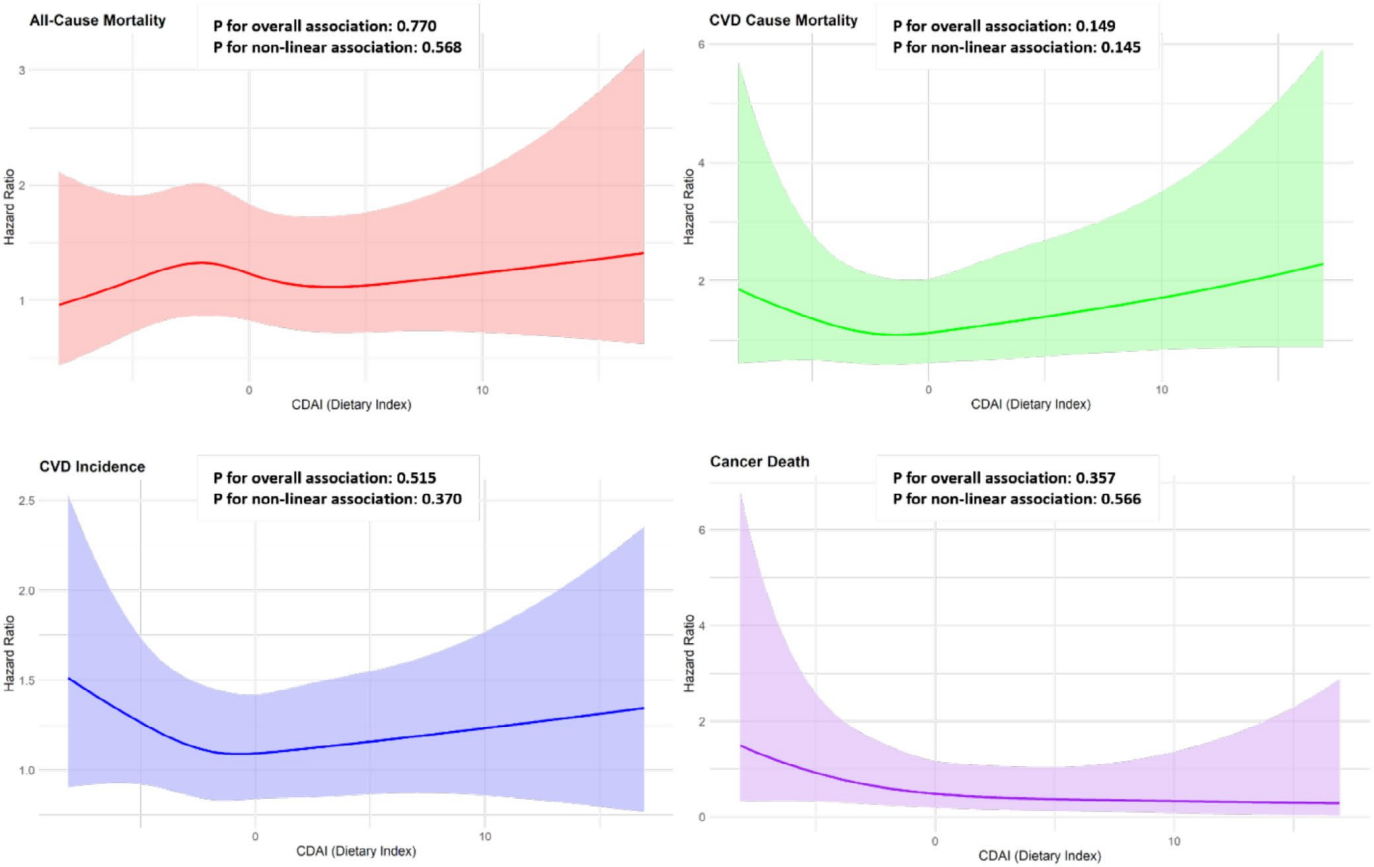
The restricted cubic spline models demonstrated distinct nonlinear relationships between CDAI and the outcomes studied, after adjusting for covariates including hypertension, diabetes, dyslipidemia, age, sex, BMI, smoking status, marital status, educational level, energy intake, physical activity, and DQI‐I.

To further explore the relationship of CDAI with mortality risk and CVD incidence, RCS analysis was performed as illustrated in Figure 2. For CVD incidence, the spline plot revealed a J‐shaped pattern, where intermediate CDAI levels were associated with the lowest risk, while both lower and higher levels showed increased risks. However, the P‐value for nonlinearity (*p* = 0.370) and the overall effect of CDAI (*p* = 0.515) indicated no significant association. For CVD‐related mortality, a U‐shaped trend was observed, with intermediate CDAI levels showing the lowest risk. However, the nonlinear effect (*p* = 0.145) and overall effect (*p* = 0.149) were again not statistically significant. For all‐cause mortality, the spline plot indicated a slight decrease in risk at lower CDAI values followed by stabilization, but both the nonlinear (*p* = 0.568) and overall effects (*p* = 0.770) were nonsignificant. The restricted cubic spline plot illustrates the relation between the CDAI and the log hazard ratio for cancer‐related mortality, adjusted for relevant covariates. The curve exhibits a slight downward trend, suggesting a potential protective consequence of higher CDAI levels on cancer‐related mortality. However, the wide confidence intervals, particularly at the extremes of the CDAI range, indicate considerable uncertainty, likely due to sparse data in those regions. The mid‐range of the curve remains relatively stable, showing no pronounced changes in the hazard ratio. ANOVA results revealed that neither the overall effect of CDAI (*p* = 0.357) nor its nonlinear component (*p* = 0.566) is statistically significant, indicating no strong evidence for either a linear or nonlinear association.

**FIGURE 3:**
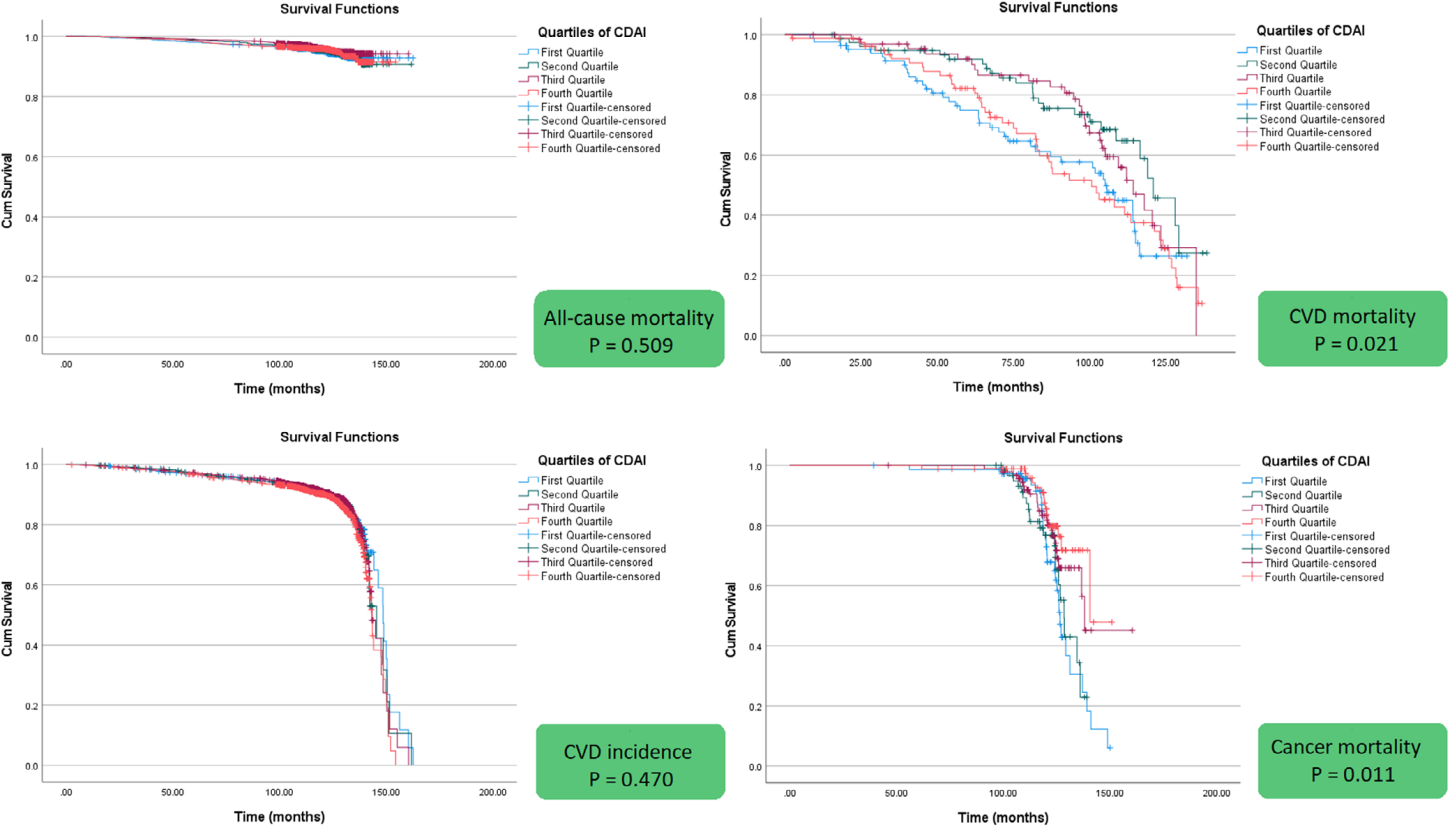
Kaplan–Meier survival curve of CDAI quartiles with outcomes.

As illustrated in Figure 3, survivals from cancer were demonstrated by weighted Kaplan–Meier survival curves based on CDAI quartiles. In comparison with the lowest quartile, Q3 and Q4 displayed significantly lower cancer cause mortality that suggests better survival outcomes. With regard to the Log‐rank tests, subjects with lower CDAI show a tendency towards a higher risk of mortality caused by cancer (*p* = 0.011). Similarly, a significant variation was observed in cardiovascular mortality between CDAI quartiles (*p* = 0.021), with Q3 and Q4 showing a reduced risk compared to Q1. No significant differences in all‐cause mortality (*p* = 0.470) and incidence of CVD (*p* = 0.509) were observed across CDAI quartiles.

**FIGURE 4:**
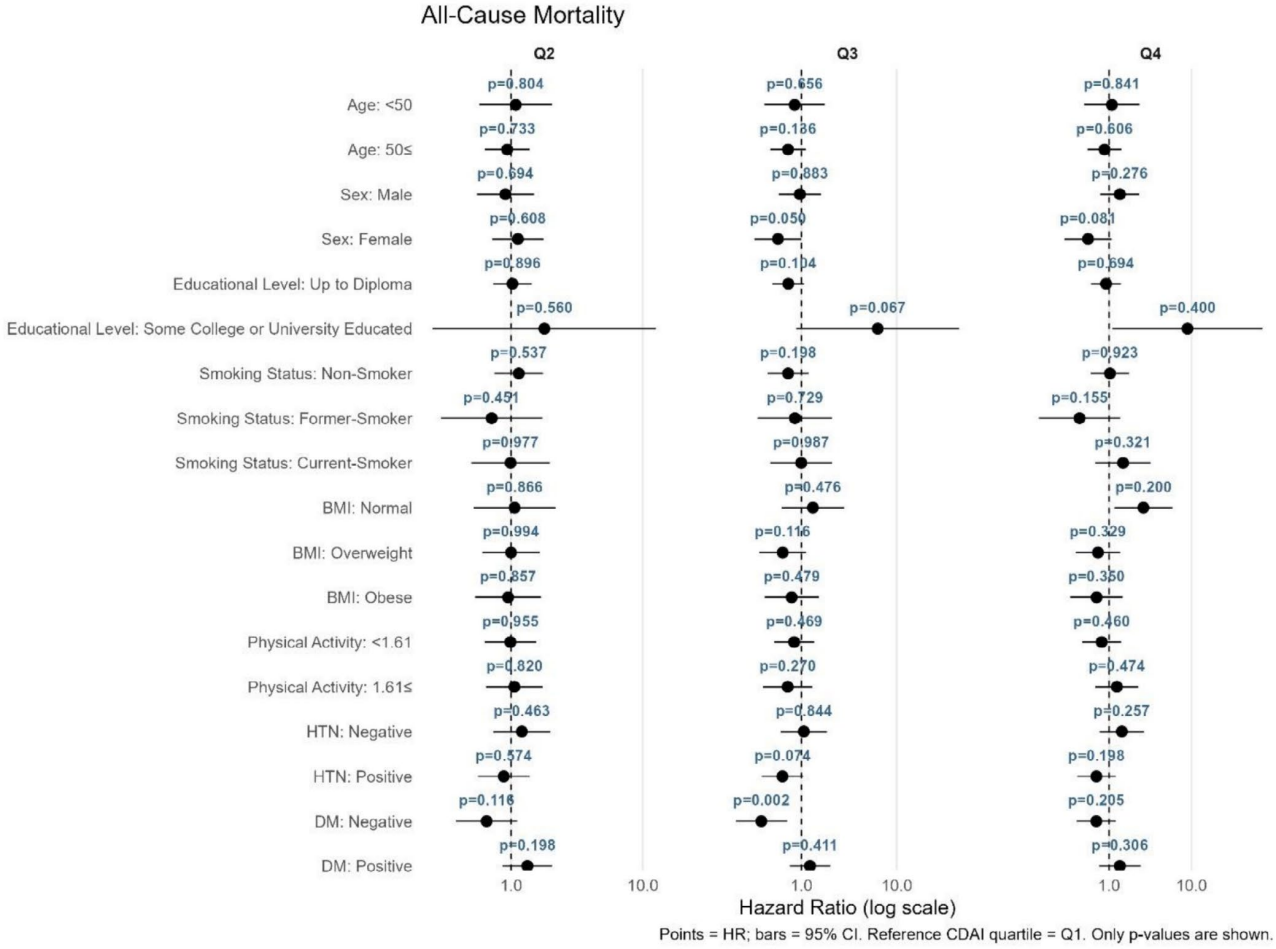
Forest plot for the association between CDAI quartiles and all‐cause mortality. Hazard ratios (HRs) and 95% confidence intervals were derived from stratified Cox proportional hazards models, using the first quartile (Q1) of CDAI as the reference.

**FIGURE 5:**
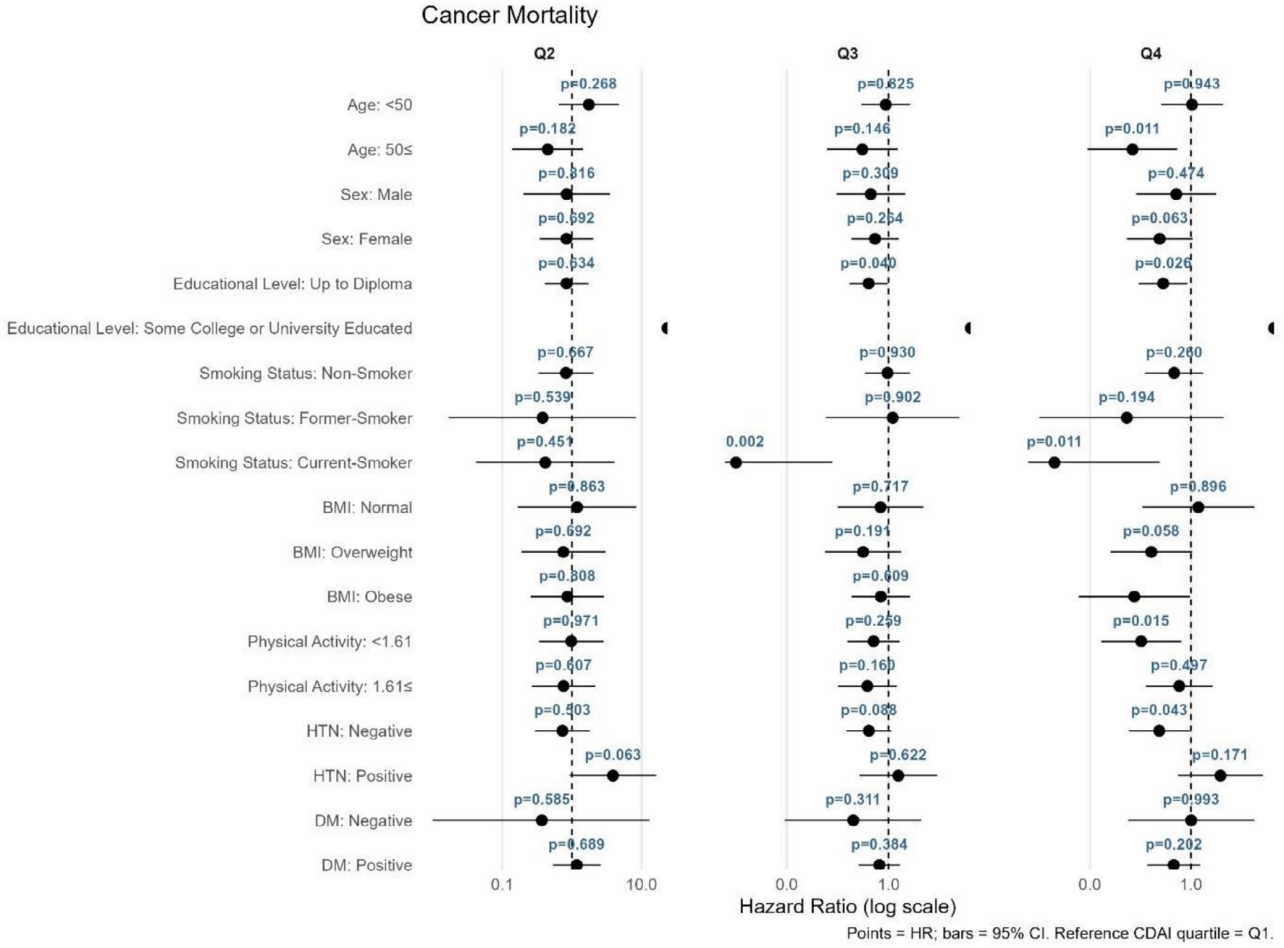
Forest plot for the association between CDAI quartiles and cancer mortality.

**FIGURE 6:**
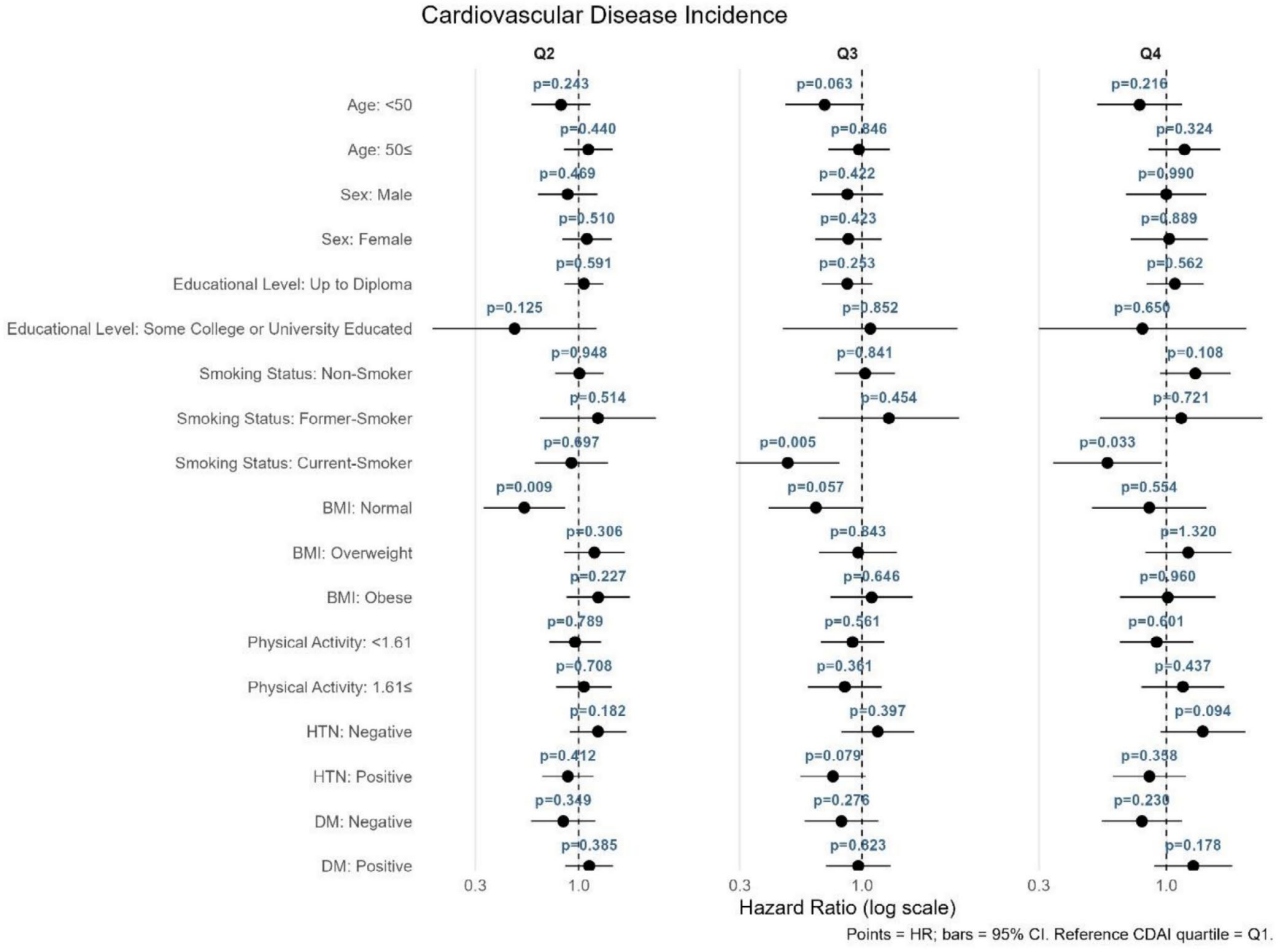
Forest plot for the association between CDAI quartiles and CVD incidence.

**FIGURE 7:**
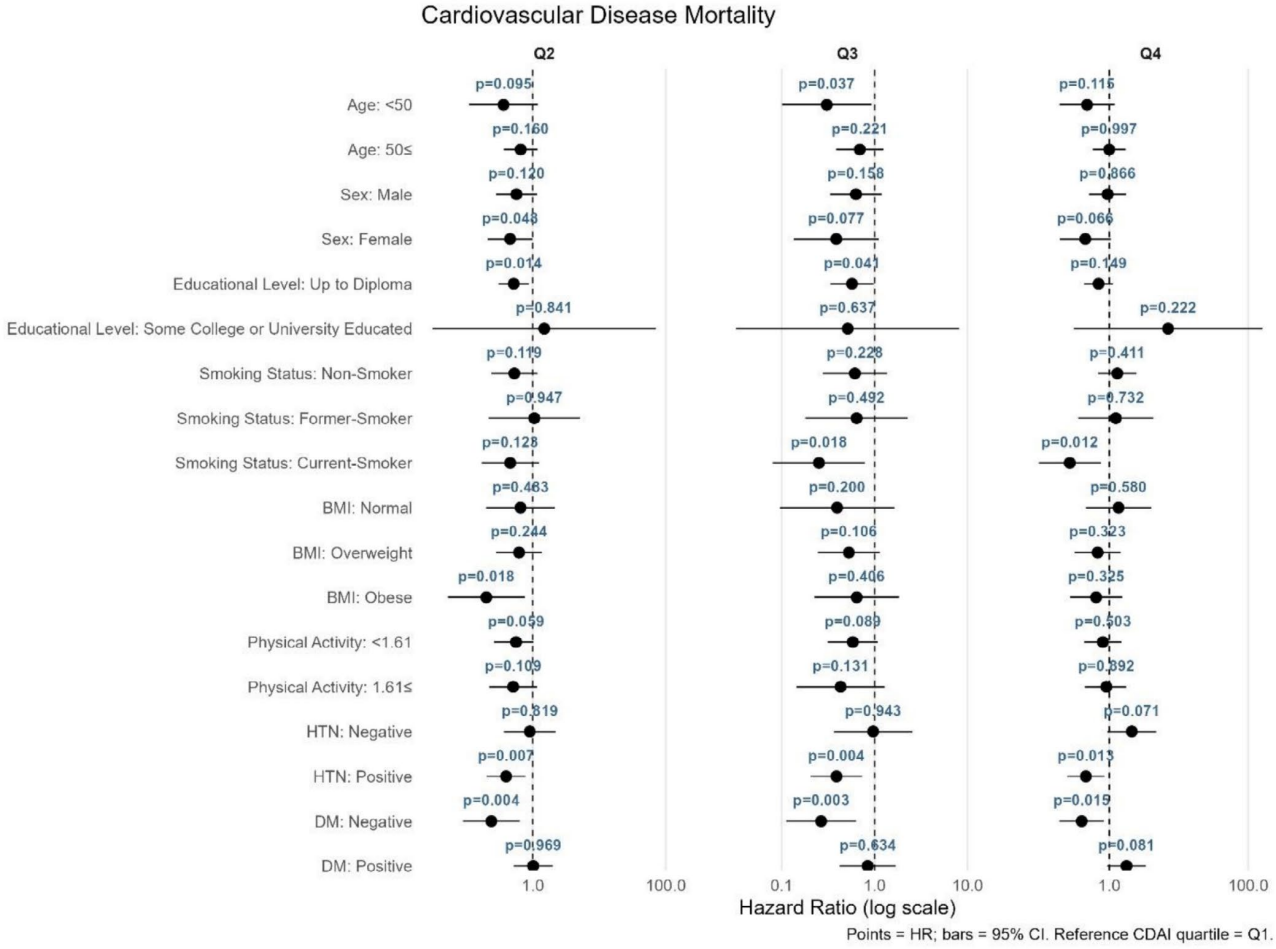
Forest plot for the association between CDAI quartiles and CVD mortality.

The relationship between CDAI quartiles and mortality or CVD outcomes was assessed using stratified Cox proportional hazards models, with the first quartile (Q1) as the reference category (Figures 4, 5, 6, 7). As shown in Figure 4 for all‐cause mortality, the comparison of Q4 versus Q1 revealed no significant association between the CDAI and all‐cause mortality in any subgroup; all findings were nonsignificant (*p* > 0.05). For cancer mortality (Figure 5), compared with Q1, significant associations were found in individuals aged ≥ 50 years (*p* = 0.011) and current smokers (*p* = 0.011). Additionally, a reduced risk was also observed in those with low physical activity (*p* = 0.015). For CVD incidence as illustrated in Figure 6, a significant association was observed in Q4 compared with Q1 among current smokers (*p* = 0.033). For CVD mortality (Figure 7), several subgroups showed significant associations in Q4 compared with Q1, including individuals with hypertension (*p* = 0.013), those without diabetes (*p* = 0.015), and current smokers (*p* = 0.012).

**FIGURE 8:**
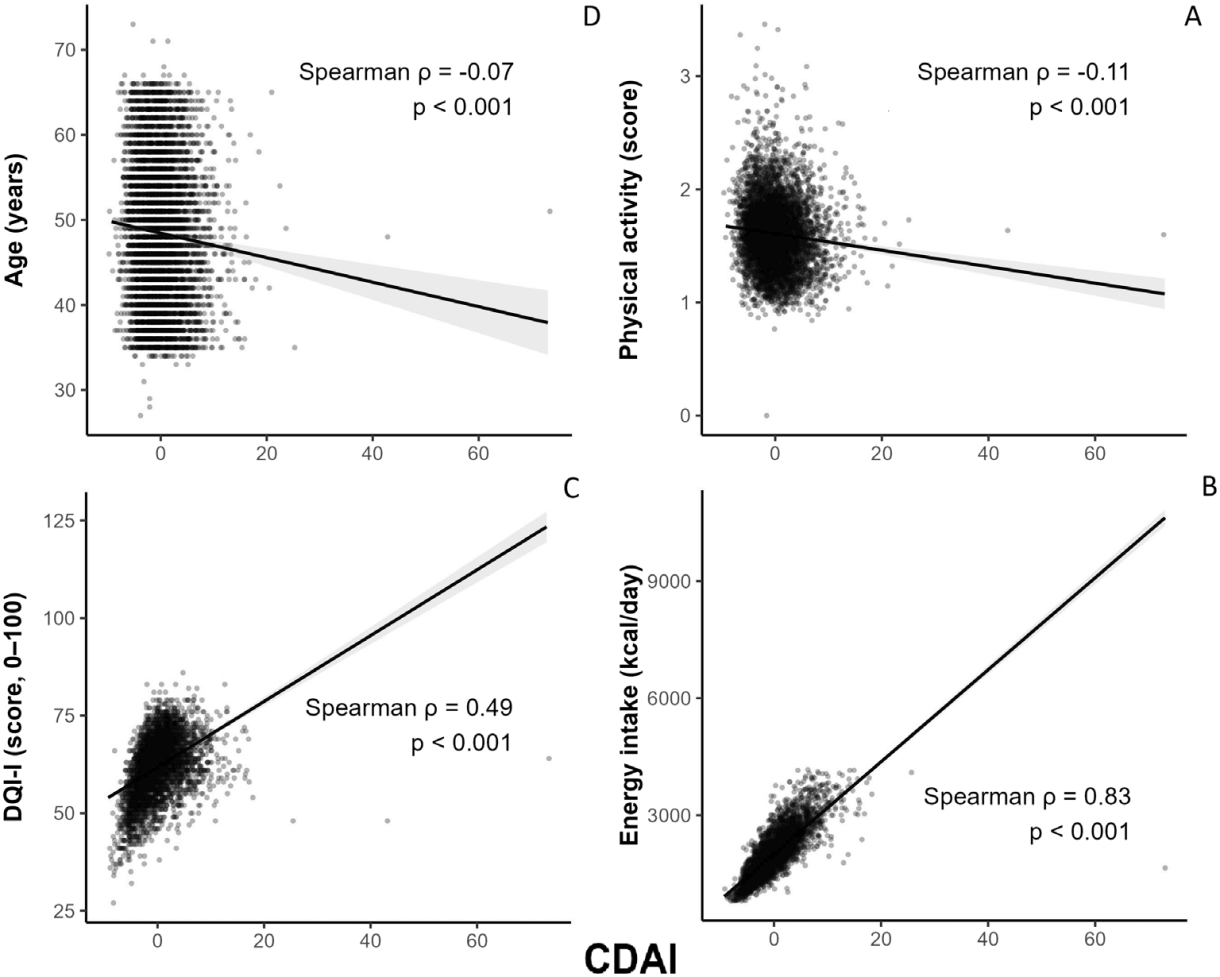
Scatter plots of the CDAI against key variables. Spearman's rank correlation coefficients (*ρ*) and *p*‐values are shown for the relationships between CDAI and (A) physical activity level, (B) energy intake (kcal/day), (C) DQI‐I score, and (D) age.

A scatter plot in Figure 8, illustrated the correlations of the CDAI and key variables that had shown significant differences across its quartiles. Spearman correlation analysis revealed statistically significant associations between the CDAI and both the DQI‐I score (*ρ* = 0.49, *p* < 0.001) and energy intake (*ρ* = 0.83, *p* < 0.001). Also, a significant negative correlation was observed between the CDAI and physical activity level (*ρ* = −0.11, *p* < 0.001) and age (*ρ* = 0.07, *p* < 0.001).

## 4 Discussion

Results of the present study revealed that higher CDAI was associated with a reduced risk of both cardiovascular and cancer‐related mortality. However, no significant association was observed between CDAI and the incidence of CVD or all‐cause mortality. Kaplan–Meier analysis further supported these findings, indicating that individuals with higher CDAI scores experienced improved survival outcomes for both cancer and cardiovascular conditions.

Several studies have worked on the association between CDAI and mortality resulting from all causes, cardiovascular mortality, and incidence with inconsistent results (Qin et al. 2024; Ma, Liu, et al. 2023; Sun et al. 2025). A study that examined the relationship between CDAI and heart failure risk found a negative association with the prevalence of heart failure, but this study did not report cardiac mortality specifically (Ma, Liu, et al. 2023). Also, another study conducted on adults with hypertension found that the top quartile of CDAI was related to a 23% fall in all‐cause mortality. The investigation found that higher CDAI was associated with a 17% lower risk of cardiovascular mortality. This finding, however, was not statistically significant (Qin et al. 2024). These results are in line with those of the present study, which suggest a U‐shaped association between CDAI and cardiovascular mortality, with significant risk reduction observed in the second and third quartiles, and a nonsignificant 15% reduction in the highest quartile. Another study on chronic kidney disease (CKD) sufferers similarly found an inverse association between CDAI and all‐cause as well as cancer mortality. Nevertheless, no significant interrelation was observed between CDAI and cardiovascular mortality after adjustment for confounding variables (Sun et al. 2025). Variations in results could be due to other factors, inclusive of differences in the study population (e.g., healthy individuals vs. patients with chronic diseases), methods of measuring CDAI, length of follow‐up, and the adjustments made for confounding factors.

The findings of the present study on cancer mortality showed that higher CDAI was related to a significantly lower cancer‐related mortality in the crude and semi‐adjusted models, especially in the second and third quartiles. However, this correlation was not statistically significant in the final model. There are several studies on the relation between dietary antioxidant intake and cancer mortality. As for CKD patients, the risk of cancer mortality fell by 36% in the top CDAI quartile when contrasted with the quartile positioned at the lowest level. A nonlinear (J‐shaped) interrelation was also detected between CDAI and cancer mortality (Sun et al. 2025). In patients suffering from chronic obstructive pulmonary disease (COPD), an increase in each unit of CDAI was correlated with a 9% decrease in cancer‐ caused mortality, and the group with the highest CDAI had a 61% reduction in risk (Li et al. 2025). Additionally, in another study on adults with hypertension, the risk of cancer mortality was 36% lower in the top quartile of CDAI than that of the quartile at the lowest level, and a similar nonlinear relationship was observed (Qin et al. 2024). In the general population, the combined intake of antioxidants has been examined with cancer mortality with conflicting results (Aune et al. 2018; Serafini et al. 2012). Some research has shown an inverse interrelation (Wright et al. 2004; Yu et al. 2022), while other investigations have pointed to no significant association (Russnes et al. 2016; Sheng et al. 2022). In the present study, CDAI was also connected to a 53.1% declined prospect of cancer mortality, although it was not statistically significant. The resulting differences could be related to methodological differences, such as how antioxidant intake was assessed and the variation in demographic characteristics in each study. Since a single antioxidant might not likely reflect an individual's sum of taken antioxidants (Sasanfar et al. 2021), the CDAI measurement was considered to fill this gap.

The observed association of CDAI with CVD‐related mortality and cancer can be explained by its anti‐inflammatory and antioxidant effects. Inflammation and oxidative stress are definitely thought to lead to endothelial cell damage and smooth muscle dysfunction, which ultimately contribute to the development of CVD (Abaj et al. 2021; Ye et al. 2023). It is widely known that antioxidant foods can exhibit antioxidant activities and regulate gene expression by reducing reactive oxygen species (ROS) and using molecules to diminish the negative effects of oxidative stress (An et al. 2022). Selenium and zinc are key antioxidants working contrary to oxidative stress, especially selenium, which prevents lipid peroxidation and oxidative stress‐incurred cell damage by binding to selenoproteins (Barchielli et al. 2022). Increased oxidative stress and inflammation are also usually observed in cancer (Reuter et al. 2010). Although some studies conducted recently have suggested that reactive oxygen species (ROS) may have an antitumorigenic role, others believe that antioxidants may act protumorigenically (Wang et al. 2016; Wiel et al. 2019). However, many recent studies have shown that antioxidants shield tumorous cells from ROS‐induced DNA destruction, which can lead to tumor cell proliferation (Balkwill 2009; LuuHung 2015). In addition, antioxidants, as anti‐inflammatory agents, play a critical role in the formation of tumors. Therefore, CDAI, as an index with anti‐inflammatory and antioxidant characteristics, can be effective in reducing mortality from CVDs and cancer by reducing oxidative stress and inflammation (Ma, Zhou, et al. 2023; Wang et al. 2023).

Furthermore, our subgroup analyses using forest plots revealed that the association between the CDAI and cause‐specific mortality was not uniform but was significantly modified by individual characteristics. The most pronounced protective associations against both cancer and cardiovascular mortality were observed among current smokers, suggesting that the beneficial effects of dietary antioxidants are particularly critical in populations exposed to high levels of exogenous oxidative stress (Feng et al. 2025). This finding was reinforced by a similarly significant association in individuals with hypertension, further supporting the role of antioxidants in mitigating oxidative stress‐related pathophysiology (Villaverde et al. 2019; Zhang et al. 2025). Additionally, while a higher CDAI was linked to a reduced risk of cancer mortality, this association was primarily significant in adults older than 50 years, a finding that may be attributed to the cumulative nature of oxidative damage, the increasing prevalence of age‐related metabolic alterations, coupled with potential changes in dietary patterns and antioxidant requirements in this age group (Ames 2018; Kong et al. 2022; Jin et al. 2025).

This study was designed using high‐quality data and long‐term follow‐up. While medical research typically focuses on medications and clinical intervention, our study underscores the notable import of dietary antioxidants in lowering the risk of death from cancer and heart disease. Our analysis reveals the complex bonding link between dietary antioxidants and mortality and provides a more comprehensive understanding for preventive measures. Given the rising prevalence of deaths from cancer and CVDs, studies targeting these groups are of great importance. The study also provides guidance for future nutritional interventions by examining the role of antioxidants in reducing mortality risk and allows medical experts and patients to prescribe and opt for more educated dietary choices. Ultimately, our research emphasizes the critical role of diets enriched by antioxidants in lowering overall mortality and those caused by other specific reasons and thus contributes to improving healthcare strategies.

Our study suffers certain limitations, too. First of all, bearing in mind the nature of observational cohort studies, we are only able to analyze the correlation between intakes of dietary antioxidants and mortality in adults and cannot determine causality. A second consideration is that employing self‐reported data in the diet and disease status questionnaires may be associated with bias and differences between actual conditions and what was reported. Third, because this study only included Iranian adults, subgroup analyses in particular for differing ethnicities or disparate populations were not possible. Fourth, although the CDAI is a useful tool, it is limited to six specific antioxidants and does not account for other potent compounds such as polyphenols, which are abundant in the Iranian diet and may contribute significantly to the overall antioxidant capacity.

## 5 Conclusions

In conclusion, this study highlights the significant relationship between the Composite Dietary Antioxidant Index and cardiovascular and cancer mortality, particularly in the middle CDAI quartiles. Our findings imply that higher antioxidant intake, reflected by CDAI, may lessen cardiovascular mortality, while the relationship with cancer mortality became less significant after adjustment for confounding factors. Future research is called for to confirm the benefits of antioxidant‐rich diets in declining the occurrence of cardiovascular diseases and cancer, providing valuable insights for public health strategies and nutritional interventions.

## Author Contributions

**Niloufar Abdollahpour:** writing – original draft (equal). **Nadia Homayounfar:** writing – original draft (equal). **Farima Farsi:** formal analysis (equal). **Sara Saffar Soflaei:** data curation (equal). **Mohammad Kalate Rahmani:** investigation (equal). **Mina Mousavi:** investigation (equal). **Mohsen Mouhebati:** conceptualization (equal). **Habibollah Esmaily:** formal analysis (equal). **Najmeh Seifi:** conceptualization (equal), methodology (equal), supervision (equal), writing – review and editing (equal). **Majid Ghayour‐Mobarhan:** conceptualization (equal), project administration (equal), supervision (equal), writing – review and editing (equal).

## Funding

This study was supported by Mashhad University of Medical Sciences (MUMS). The funders were not involved in study design; collection, analysis, and interpretation of data; in the writing of the report; and in the decision to submit the article for publication.

## Ethics Statement

All experiments were performed in accordance with the declaration of Helsinki protocol and Mashhad University of Medical Sciences ethical guidelines and regulations. The research protocol was approved by the School of Medicine, Mashhad University of Medical Sciences, Biomedical Research Ethics Committee (IR.MUMS.MEDICAL.REC.1395.458). All participants signed a written informed consent before participating in the study.

## Consent

The authors have nothing to report.

## Conflicts of Interest

The authors declare no conflicts of interest.

## Data Availability

The datasets generated and/or analyzed during the current study are not publicly available due to university data ownership policies, but are available from the corresponding author (Prof. Majid Ghayour‐mobarhan) on reasonable request.
